# Sialometabolism in Brain Health and Alzheimer’s Disease

**DOI:** 10.3389/fnins.2021.648617

**Published:** 2021-03-30

**Authors:** Punam Rawal, Liqin Zhao

**Affiliations:** ^1^Department of Pharmacology and Toxicology, School of Pharmacy, University of Kansas, Lawrence, KS, United States; ^2^Neuroscience Graduate Program, University of Kansas, Lawrence, KS, United States

**Keywords:** sialic acid, sialylation, ganglioside, neural cell adhesion molecule, PSA-NCAM, Siglec, CD33, late-onset Alzheimer’s disease

## Abstract

Sialic acids refer to a unique family of acidic sugars with a 9-carbon backbone that are mostly found as terminal residues in glycan structures of glycoconjugates including both glycoproteins and glycolipids. The highest levels of sialic acids are expressed in the brain where they regulate neuronal sprouting and plasticity, axon myelination and myelin stability, as well as remodeling of mature neuronal connections. Moreover, sialic acids are the sole ligands for microglial Siglecs (sialic acid-binding immunoglobulin-type lectins), and sialic acid-Siglec interactions have been indicated to play a critical role in the regulation of microglial homeostasis in a healthy brain. The recent discovery of CD33, a microglial Siglec, as a novel genetic risk factor for late-onset Alzheimer’s disease (AD), highlights the potential role of sialic acids in the development of microglial dysfunction and neuroinflammation in AD. Apart from microglia, sialic acids have been found to be involved in several other major changes associated with AD. Elevated levels of serum sialic acids have been reported in AD patients. Alterations in ganglioside (major sialic acid carrier) metabolism have been demonstrated as an aggravating factor in the formation of amyloid pathology in AD. Polysialic acids are linear homopolymers of sialic acids and have been implicated to be an important regulator of neurogenesis that contributes to neuronal repair and recovery from neurodegeneration such as in AD. In summary, this article reviews current understanding of neural functions of sialic acids and alterations of sialometabolism in aging and AD brains. Furthermore, we discuss the possibility of looking at sialic acids as a promising novel therapeutic target for AD intervention.

## Sialic Acid Structure and Metabolism: An Overview

Sugars have diverse physiological functions beyond serving as a source of energy via cellular respiration or as a component of cell wall polysaccharides and the nucleic acid backbone (Gabius and Roth, 2017). The extent of structural and compositional variability offered by sugars is unsurpassed in nature. Mono-, oligo-, or polysaccharides attached proteins or lipids are termed as glycoconjugates. Sugars of the glycoproteins and glycolipids are commonly referred to as glycans (Taylor and Drickamer, 2011), and they are mostly terminated with sialic acids, an acidic sugar unit with a 9-carbon backbone (Varki, 2008; Schnaar et al., 2014). The process of covalent addition of sialic acid to glycoconjugates is termed sialylation (Li and Ding, 2019).

Sialic acid (also known as neuraminic acid) nomenclature originated from its discovery (Siddiqui et al., 2019). The Swedish chemist Gunnar Blix first isolated it from salivary mucins and called it “sialic acid” after the Greek word for saliva. The same substance was then independently discovered by German scientist Ernst Klenk in 1941 in brain glycolipids and named “neuraminic acid” to relate to neural tissue, the source tissue in which it was found. Structurally, sialic acid or sia refers to any of the identified 50 members of the family of neuraminic acid (Neu, 5-amino-3,5-dideoxy-D-glycero-D-galacto-non-2-ulosonic acid) (Karim and Wang, 2006). Neu represents C-5 free amine form and is rarely encountered in nature. More prevalent sialic acids are N-acetyl and N-glycolyl derivatives of Neu commonly referred to as Neu5Ac and Neu5Gc, respectively (Schnaar et al., 2014). The term sialic acid is generally used to refer to Neu5Ac, the most abundant sialic acid in humans (Schauer, 2004).

Cytosolic conversion of a nucleotide sugar UDP-N-acetylglucosamine (UDP-GlcNAc) to N-acetyl-D-mannosamine (ManNAc) and subsequently to N-acetyl-D-mannosamine 6-phosphate (ManNAc-6-P) by a bifunctional enzyme GNE (UDP-GlcNAc 2-epimerase/ManNAc kinase) are the first steps in the biosynthesis of sialic acid in mammals (Schwarzkopf et al., 2002; Li and Chen, 2012). Condensation of ManNAc-6-P with phosphoenolpyruvate (PEP) by N-acetylneuraminic acid 9-phosphate synthase forms N-acetylneuraminic acid 9-phosphate (Neu5Ac-9P). Neu5Ac-9P dephosphorylation by N-acetylneuraminic acid-9-phosphate phosphatase gives rise to free sialic acids in the cytoplasm, mainly Neu5Ac. In the nucleus, sialic acids are then converted to their activated nucleotide form (CMP-Sia) by CMP-Sia synthases using cytidine triphosphate (CTP) as a donor. CMP-Sia then returns to the cytoplasm and is further translocated into the lumen of Golgi apparatus via an antiporter in exchange for CMP. Sialylation occurs when a newly synthesized glycoconjugate is terminated by sialic acid during its passage through the golgi compartment by sialyltransferase (ST) (Li and Chen, 2012). The enzyme mediates the attachment at C-2 carbon of sialic acid via one of the following linkages: α 2–3 or α 2–6 to galactose (Gal), α 2–6 to N-acetylglucosamine (GlcNAc) or N-acetylgalactosamine (GalNAc), or α 2–8 when bound to another sialic acid (Harduin-Lepers et al., 2005). Twenty mammalian STs have been identified and comprise four groups: ST3Gal, ST6Gal, ST6GalNAc, and ST8Sia based on their primary substrates, Gal, GalNAc, and Sia, as well as the linkage generated (α2–3, α 2–6, or α 2–8) (Schnaar et al., 2014).

Removal of sialic acid from a sialoglycan is mediated by sialidase also known as neuraminidase, which is found in lysosomes, on the cell surface or in cytoplasm (Varki and Schauer, 2009). The regenerated sialic acids can be further utilized in the sialylation of glycoconjugates. There are 4 different types of neuraminidases in humans, NEU1-NEU4 (Giacopuzzi et al., 2012; Monti and Miyagi, 2012). The most abundant sialidase is NEU1 expressed in the lysosome, which is responsible for removing sialic acid from oligosaccharides and glycoproteins with no action on gangliosides. NEU2 is a cytosolic sialidase involved in the removal of sialic acid from a wide variety of glycan structures. NEU3 is localized on the cell plasma membrane and specifically desialylates gangliosides. NEU4 is primarily found on intracellular membranes with a broad range of glycan specificity. Studies have identified and implied various physiological roles for these neuraminidases. NEU1 gene mutation has been linked to a congenital lysosomal storage disorder called sialidosis that affects the nervous system in humans (Seyrantepe et al., 2003). NEU1 has also been shown to be upregulated and localized to plasma membrane in activated T-cells (Nan et al., 2007) and differentiating monocytes (Liang et al., 2006). Furthermore, NEU1-NEU3 are found to be important for skeletal muscle differentiation (Fanzani et al., 2012).

## Sialic Acid Physiochemical and Biological Properties

The location and ubiquitous distribution of sialic acid allows it to mediate a diverse range of physiological and pathological processes (Varki, 2008). Sialic acid functions can be categorized into two types. The first is more of a general role because of its charge and hydrophilicity. This function primarily affects physiochemical properties of the underlying glycoconjugates (Schauer et al., 1995). Negatively charged sialic acid on human erythrocytes provides charge repulsion and prevents unwanted cellular interactions in the blood (Born and Palinski, 1985). An unusually high concentration of sialic acid has also been reported on the luminal surfaces of vascular endothelia (Varki, 2008). This results in a mutual repulsion between blood cells and endothelial surfaces and prevents impediment of circulation. In addition to its role in the circulatory system, the importance of sialic acid in the renal system has also been recognized. Sialic acid is expressed in foot processes of podocytes, a specialized group of cells in kidney glomerulus. Podocytes prevent entry of plasma proteins into urinary ultrafiltrate by forming a barrier consisting of filtration slits. The polyanionic nature of podocyte epithelial cells that sialic acid offers maintains the slit integrity (Dekan et al., 1991). Intraperitoneal injection of sialidase to remove sialic acid was found to cause proteinuria and renal failure in a dose dependent manner in mice (Gelberg et al., 1996). Therefore, sialic acid is essential for normal and efficient kidney filtration function. Sialic acid also lines the epithelial border of airways and is a primary component of mucins, the building blocks for mucus (Schauer et al., 1995). Sialic acid contributes to the anionic and hydrophilic properties of mucins and thus maintains the required rheological activities of mucus to lubricate airways and trap pathogens from inhaled air.

The second category of sialic acid functions is more specific and deals with cellular and molecular recognition (Schauer et al., 1995). This can either mask or facilitate biological identification, allowing sialic acid to exert dual and opposite effects. Binding and uptake of desialylated glycoproteins by hepatocytes is a well-characterized example of the masking function (Harford et al., 1984). In the absence of sialic acid, underlying glycoproteins are recognized by receptors in organs such as the liver and are rapidly cleared (Weigel and Yik, 2002). Therefore, sialic acid plays an important role in determining half-lives of circulating glycoproteins (Raju et al., 2001). For this reason, therapeutic glycoproteins are increasingly being synthesized with sialic acid capping to prolong their serum half-lives (Raju et al., 2001). In addition to increasing molecular life span, sialic acid can also promote cell survival in a pathological condition like cancer. Sialic acid expression is altered during the period of tumor transformation and malignant progression (Zhou et al., 2020). Hypersialylation is commonly reported in different types of cancers such as leukemia, ovarian cancer, colorectal cancer, and breast cancer. Mounting evidence show that sialic acid can downregulate host immune activation to cancer cells (Rodrigues and Macauley, 2018). Sialic acid-binding immunoglobulin-type lectin (Siglec) is the major mediator through which inhibitory signals are transmitted from sialic acid to the immune system. Siglecs are type-1 membrane proteins containing an extracellular region with Ig-like domains and a homologous V-set domain responsible for sialic acid recognition. The V-set domain has an arginine residue that is critical for forming a salt bridge with negatively charged sialic acids (Pillai et al., 2012). Cytoplasmic domains of most of the Siglecs possess several immunoreceptor tyrosine-based inhibitory motifs (ITIMS) and ITIM-like signaling motifs. ITIMs are phosphorylated by Src family kinases upon ligand binding on tyrosine residues. Following phosphorylation, ITIMs recruit Src homology domain-2 phosphatase-1 (SHP-1) and SHP-2 (Crocker and Varki, 2001). SHP-1 has been shown to dephosphorylate a variety of signaling molecules in immune cells and reduce the release of inflammatory mediators such as nitric acid (NO), tumor necrosis factor-alpha (TNF-α), and interleukin-1 beta (IL-1β) (Zhao et al., 2006). The reduction of inflammatory response may be mediated by inhibiting MAP kinase activity (Paulson et al., 2012; Pillai et al., 2012). Thus, sialic acid-Siglec interaction causes the immune system to switch to an “off” state, resulting in immune evasion and cancer progression. Strategies for interrupting this interaction are emerging in the field of cancer therapeutics (Rodrigues and Macauley, 2018).

**FIGURE 1:**
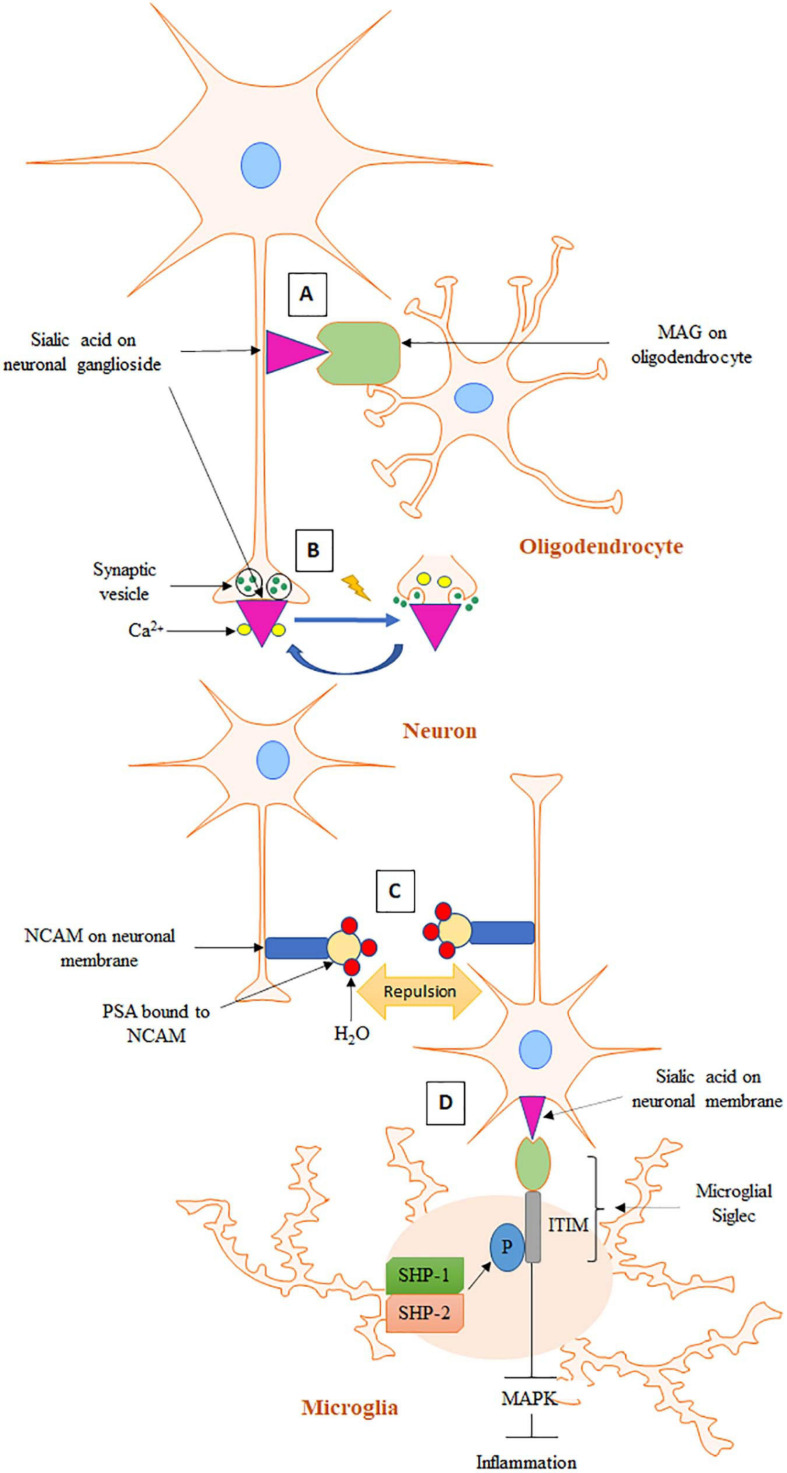
Schematic representation of sialic acid functions in the brain. **(A)** In the process of axon myelination, MAG on oligodendrocyte interacts with sialic acid of ganglioside on axonal membrane. **(B)** At resting state, Ca^2+^ is bound to negatively charged sialic acid on synaptic membrane. In the event of an action potential, Ca^2+^ is released from sialic acid and enters presynapse for exocytosis and neurotransmitter release. **(C)** PSA on NCAM causes stereochemical repulsion that prevents homophilic contacts of NCAMs on opposing cells. **(D)** Sialic acid binds to microglial Siglec, which induces phosphorylation of ITIM present on the cytosolic domain of Siglec. Phosphorylated ITIM recruits SHP-1 and SHP-2 leading to inhibition of MAPK activity and the subsequent inflammatory cascade.

Sialic acid recognition can also facilitate many biological processes (Varki, 2008). It has been well established that influenza virus hemagglutinin can recognize and bind to sialic acids on the airway epithelium. Hemagglutinin is a glycoprotein expressed on the surface of influenza virus. Attachment of hemagglutinin to sialic acid causes host cell internalization of the virus. Sialic acid thus plays a critical role in the first step of influenza virus infection. In addition, sialic acid-mediated recognition is also well documented in the brain, an organ with the highest levels of sialic acid in the body (Schnaar et al., 2014). Sialic acid in the brain is most abundantly expressed in gangliosides (65%), followed by glycoproteins (32%) and less than 3% in free form (Proia, 2004). Gangliosides are amphipathic molecules made up of a ceramide lipid anchor attached to an oligosaccharide chain of variable length. They are localized on the surface of mammalian cells and are most abundant on neuronal cell surfaces. Although a wide variety of ganglioside structures have been detected, four closely related ganglioside structures, GM1, GD1a, GD1b, and GT1b, together represent 97% of gangliosides present in an adult human brain and are referred to as “complex gangliosides” (Tettamanti et al., 1973). Interactions between sialic acids on complex gangliosides and Siglecs are crucial for axon myelination (Weinhold et al., 2005). The mechanism for the interaction is discussed in detail in section “Sialic Acid and Sialylation Functions in the Brain” and Figure 1.

Human diseases associated with impaired sialic acid metabolism can lead to severe neurological defects (Boomkamp and Butters, 2008). A case study was reported of an infant suffering from severe mental deficits and ultimately death 3 months after birth (Schnaar et al., 2014). The postmortem showed a total deficiency of complex gangliosides in the brain with significantly reduced ganglioside synthase activity. Furthermore, the brain exhibited diminished myelin structures, emphasizing the crucial role of gangliosides in axon-myelin interactions. Salla disease is characterized by impaired lysosomal sialic acid storage that results in ataxia, hypotonia, delayed motor development, and seizures (Prolo et al., 2009). In addition, hypomyelination with thin corpus callosum is also usually reported. Infantile free sialic acid storage diseases (ISSD) is another lysosomal sialic acid storage disease with a more severe and debilitating pathology leading to failure of multiple organs and premature death (Schnaar et al., 2014). Both Salla disease and ISSD are caused by mutations in sialin, a lysosomal membrane exporter of sialic acid. Sialin is encoded by the *SLC17A5* gene, mutation in which causes lysosome to sequester sialic acid. As a result, free sialic acid fails to be transported out of the lysosomes into cytoplasm. A decrease in sialin activity is associated with an increased disease severity. Sialin-KO mouse models exhibited human disease phenotypes with poor coordination, seizures, tremors and early death (Prolo et al., 2009). A marked brain hypomyelination was also observed in these mouse models. A study aimed to understand the mechanism for this pathology in sialin-KO mice reported a significantly reduced number of postmitotic oligodendrocytes, glial cells responsible for axon myelination (Van Den Bosch, 2017). This decrease was associated with an increase in cell apoptosis during later stages of myelin formation. Thus, sialic acid deficiency causes an impaired maturation and increased apoptosis of oligodendrocytes leading to myelination defects. Sialin deficiency can also lead to alterations in ganglioside metabolism, thereby compromising axon myelination (Pitto et al., 1996).

## Sialic Acid and Sialylation Functions in the Brain

### Axon Myelination

Development of the term “gangliosides” to describe sialic acid-bound glycosphingolipids in the brain underscores its abundance (Schnaar, 2004). Complex gangliosides on axons serve as receptors for myelin-associated glycoprotein (MAG), a founding member of the Siglec family (Figure 1A; Crocker et al., 1998). MAG is a transmembrane protein expressed only on myelinating cells–oligodendrocytes and Schwann cells in the CNS and PNS, respectively (Kelm et al., 1994). MAG is located in the innermost wrap of myelin juxtaposed to axonal surface (Schnaar and Lopez, 2009). Because of its location, MAG was first proposed to play an important role in axon-myelin interactions. Multiple studies have since confirmed this initial hypothesis (Bartsch et al., 1997; Kossmann et al., 2007). Sialic acid-MAG interaction is required for axon myelination, which promotes axon integrity, stability, and action potential conduction velocity (Kelm et al., 1994; Schnaar and Lopez, 2009). *B4galnt1*-null mice are mouse models of complex gangliosides deficiency (Pan et al., 2005). These mice exhibited abnormal axon myelination, thinner axon diameters and progressive axon degeneration. MAG-KO mice exhibited the same phenotype as *B4galnt1*-null mice, underlining the importance of both gangliosides and MAG for axon myelination. All Siglecs recognize terminal sialic acid residues, however, they can show variations in sialic acid linkage specificity (Collins et al., 1999). Studies have shown that MAG preferentially binds to NeuAc 2–3 Gal 1–3 GalNAc trisaccharide, a terminal sequence shared by GD1a and GD1b gangliosides. GD1a and GD1b lacking this terminal structure did not bind to MAG, leading to a hypothesis that these two gangliosides are crucial partners of MAG in axon myelination. Despite these important leads, the detailed mechanism of MAG-sialic acid interaction and the resulting downstream signaling for axon myelination remains unknown (Lopez and Báez, 2018).

Complex gangliosides also enable MAG to shield axons from toxic insults (Schnaar et al., 2014). Vincristine is a neurotoxin that causes structural and functional damage to axons. Addition of a soluble MAG to neuronal cultures rescued them from vincristine mediated damage. However, addition of a mutant form of MAG failed to show such protective effects. The mutant MAG lacked the arginine residue shared among all Siglecs for sialic acid binding. Furthermore, wild-type MAG failed to show the same protective effect in neuronal culture of *B4galnt1*-null mice. Taken together, these observations indicate that gangliosides are a necessary component of healthy axon-MAG interactions. Moreover, it has been postulated that interaction of MAG with sialic acid regulates the expression and phosphorylation of neurofilaments, the most abundant cytoskeletal proteins in axons (Dashiell et al., 2002). Phosphorylation of neurofilaments increases negative charge and induces a side arm repositioning, allowing a larger neurofilament spacing. Increased neurofilament phosphorylation thus contributes to a reduced packing density and increased axon caliber. Axon caliber has a functional importance because diameter governs conduction velocity of myelinated nerve fibers (Hoffman et al., 1987). Higher axon caliber exhibits faster conduction velocity because of lower electrical resistance and rapid distribution of action potential (Costa et al., 2018). In summary, axon myelination increases axon caliber, an increase associated with a higher level of neurofilament phosphorylation and a lower neurofilament packing density. Thus, the axonal neurofilament packing density is regulated by a balance between kinases and phosphatases that catalyze the phosphorylation and dephosphorylation of neurofilaments, respectively (Dashiell et al., 2002). MAG is associated with an elevated level of kinases and increased neurofilament phosphorylation leading to a higher axon caliber. However, the role of MAG in phosphatase activity remains to be established (Naito-Matsui et al., 2017). The interaction between MAG and sialic acid may not always be beneficial (Schnaar et al., 2014). MAG has also been shown to inhibit axon regeneration, for which gangliosides serve as a mediator. Mutations in the sialic acid binding arginine residue on MAG decreased inhibition strength (Vinson et al., 2001). MAG binding to gangliosides activates intracellular GTPase RhoA, a small G-protein that controls actin and microtubule cytoskeletal assembly and disassembly (Hall and Lalli, 2010). GTPase RhoA activation regulates reorganization through growth cone collapse and inhibition of neurite outgrowth. The growth cone is a large, motile actin-supported structure located at growing ends of a developing or regenerating axon (Suter and Forscher, 1998). Crosslinking anti-ganglioside antibodies have been shown to mimic MAG-related axon outgrowth inhibition via RhoA pathway (Vinson et al., 2001). Furthermore, sialidase treatment has been found to increase axon outgrowth following CNS injury in animal models, validating the role of sialic acid in the inhibition (Yang et al., 2006; Mountney et al., 2010). In summary, ranging from axon myelination to protection to repressing regeneration, MAG’s functions have been linked to its interaction with sialic acid in the brain.

### Synaptic Development

Cell adhesion molecules are important components of synapses with well-established roles in forming and maintaining various synaptic structures during brain development (Benson et al., 2000). Neural cell adhesion molecule (NCAM) proteins can interact among themselves via homophilic contacts as well as form heterophilic contacts with other molecules such as neuron-glia cell adhesion molecule, fibroblast growth factor receptor and L1CAM to facilitate cell adhesion (Fiszbein et al., 2015). Addition of polysialic acid (PSA) is a very important posttranslational modification of NCAM that occurs during its passage though the Golgi apparatus (Schnaar et al., 2014). PSA is a large molecule made of linear homopolymers of α 2–8 linked sialic acids. PSA represents a major form of sialic acid bound to proteins in the brain with more than 95% of it bound to NCAM (Yang et al., 1994). PSA was first discovered for its role in reducing NCAM-mediated neuronal cell adhesion (Benson et al., 2000). Although the mechanism by which PSA reduces NCAM-mediated adhesion is not perfectly understood, it is postulated to involve steric hindrance. The polyanionic nature and large degree of hydration provided by PSA can significantly increase the overall size of a PSA carrier and thereby increase stereochemical repulsive forces (Figure 1C; Yang et al., 1994; Nakata and Troy, 2005). PSA-NCAM is the less adhesive form of NCAM and therefore an appropriate form for allowing synaptic/structural reorganization during brain development and under circumstances of synaptic plasticity during maturity (Cremer et al., 1997). PSA expression increases throughout the brain during the embryonic and perinatal stages in order to facilitate various brain development events. Axon pathfinding is a process of neural development in which neurons send out their axons to reach their appropriate targets. Axon pathfinding has cycles of fasciculation in which growing axons can travel, adhering together to form bundles (fasciculation), and separate and rearrange (defasciculation). These cycles are a prerequisite to forming new synaptic contacts and have been unequivocally demonstrated as a form of synaptic plasticity (Weledji and Assob, 2014). Endoneuraminidase-mediated selective removal of PSA from growing axons make them incapable of defasciculation generating pathfinding errors (Yin et al., 1995). Moreover, endoneuraminidase treatment impairs migrations of neurons in the olfactory bulb (Yoshida et al., 1999). Similar effects have also been observed in NCAM-KO mice (Schnaar et al., 2014). Adult NCAM, however, has reduced PSA expression associated with mature synapse formation with the exceptions of areas characterized by a high degree of modeling of structures such as the hippocampus, hypothalamus, dentate gyrus, and olfactory bulb (Cremer et al., 1994; Seki, 2002). Therefore, PSA on NCAM is necessary for cell migration, axon outgrowth, axon defasciculation, and target recognition. Long-lasting synaptic plasticity requires a process for downregulating the expression of adhesion proteins on neuronal surfaces to promote process rearrangement (Fiszbein et al., 2015). PSA present on NCAM serves as the necessary modulator for such rearrangement. Based on these findings, PSA-NCAM has been recognized as a promoter of synaptic plasticity in the brain.

### Synaptic Transmission

Sialic acids on gangliosides interact with Ca^2+^ ions via electrostatic interactions at synapse and facilitate well-regulated release of neurotransmitters (Rahmann et al., 1976; Rahmann, 1995). At resting state, Ca^2+^ ions are tightly bound by the negatively charged sialic acids at synapse and are only released when an action potential arrives at the presynaptic terminals (Figure 1B). Arrival of the action potential causes alterations in ionic concentrations and/or electric field strength, causing gangliosides to rearrange and thereby release Ca^2+^. The released Ca^2+^ can then enter the nerve terminal through voltage gated Ca^2+^ channels (Mochida, 2019). Increased Ca^2+^ levels at the presynapse can then trigger release of neurotransmitters from synaptic vesicles. Following release of the neurotransmitters, Ca^2+^ ions re-attach to the gangliosides as the resting potential is restored via ganglioside-modulated Ca^2+^-ATPase. Therefore, sialic acid on gangliosides plays an important role in mediating neurotransmission.

### Microglial Homeostasis

Microglia are brain resident myeloid cells that protect the brain from pathogenic invasions and maintain brain homeostasis and plasticity (Long and Holtzman, 2019). Microglial activation can generate anti-inflammatory and immunosuppressive signals and exert protective functions. However, it can also produce pro-inflammatory mediators such as reactive oxygen species, TNF-α, and NO, leading to neuronal damage and cytotoxicity (Hansen et al., 2018). In an acute event, a balance between microglial activation and inflammation is maintained (Spangenberg and Green, 2017). However, this balance is disrupted in neurodegenerative diseases such as AD, leading to chronic neuroinflammation. Therefore, modulation of microglial activation is crucial for retaining microglial homeostasis and reducing neuroinflammation. Microglia have been found to express a number of Siglecs including CD33, Siglec-11, and Siglec-16 in humans and CD33, Siglec E, F, and G in mice (Griciuc et al., 2013). Interaction of sialic acid with Siglec allows microglia to return to an “off” state (section “Sialic Acid Physiochemical and Biological Properties” and Figure 1D). This is supported by a study where stimulation of Siglec-11, a CD33-related Siglec, in murine microglia by cross-linking caused a decrease in phagocytosis of apoptotic neurons (Wang and Neumann, 2010). The stimulation also prevented lipopolysaccharide (LPS)-induced transcription of proinflammatory mediators IL-1β and nitric oxide synthase-2. Moreover, Siglec-11 on microglia was found to bind to PSA-NCAM in co-cultures of microglia and neurons and protected neurons from microglia-induced toxicity. However, this protective effect was not observed when PSA was removed by endoneuraminidase treatment (Wang and Neumann, 2010). Siglec E expressed on murine microglia was also found to inhibit phagocytosis of neuronal debris and eliminated the resulting oxidative burst and proinflammatory effects (Siddiqui et al., 2019). This neuroprotective effect of Siglec E was abolished upon sialidase treatment emphasizing the role of sialic acid-Siglec interaction in microglial activity regulation. Mouse Siglec F is a paralog of human Siglec-5 that binds to sialic acids in neurons (Wielgat and Braszko, 2012). Treatment of the microglia-neuron co-culture with endoneuraminidase and α-neuraminidase prevented the binding and promoted microglial activation and phagocytosis of neurons leading to increased production of microglial proinflammatory mediators such as IL-1β and nitric oxide synthase 2 and reduced neurite and neuronal cell bodies. Furthermore, a pool of PSA-containing proteins, E-selectin ligand 1 (ESL1), and neuropilin 2 (NRP2), have been identified in the golgi compartment of stem cell-derived and primary murine microglia and THP-1 human macrophages (Werneburg et al., 2016). These proteins were found to be synthesized in response to injury-induced microglia activation in adult mice brain slices (Thiesler et al., 2020). In addition, inflammatory activation by LPS induced the release of PSA-bound ESL1 and NRP2 from the golgi to extracellular space in BV2 microglia cells. Based on these observations, it was hypothesized that the increased secretion of microglia-intrinsic pool of PSA-containing proteins could serve as a negative feedback regulator of microglia activation. Consistently, Siglec E receptor expression was also significantly increased in LPS-treated BV2 cells while CRISPR/Cas9-mediated Siglec E removal prevented the protective effect of exogenously added PSA against LPS (Thiesler et al., 2020). This is further supported by a finding where LPS-induced microglial inflammation was found to be exacerbated in ST8SIA4-deficient mice, a model for impaired production and release of PSA (Werneburg et al., 2015). Taken together, these studies indicate that sialic acid in general, whether it is localized on neuronal membrane or released from microglia itself or added exogenously, provides negative feedback inhibition of microglia activation and inflammation and protects neurons under neuroinflammatory conditions.

## Sialylation in Brain Aging

In light of the crucial role of PSA-NCAM in the formation of neuronal circuitry (section “Synaptic Development”), follow-up studies have been done to analyze whether PSA is re-expressed in mature nervous systems to accommodate structural reorganization of neuronal connections (Rønn et al., 2000). Covault and Sanes reported PSA-NCAM reappearance in denervated skeletal muscles during the process of regeneration of lesioned motor neurons (Covault and Sanes, 1985). Consistent with this study, regenerating fibers were found to re-express PSA-NCAM in hippocampal organotypic cultures, which allowed for the formation of functional synaptic contacts between CA3-CA1 neurons across the sections (Muller et al., 1994). Furthermore, treating the sections with Endo-N, an enzyme that causes specific cleavage of PSA from NCAM, led to a less effective and functionally slower recovery from the lesion. These findings indicate that regeneration of both the central and peripheral neuronal fibers involves an increase in the levels of PSA-NCAM. However, the ability of PSA-NCAM re-expression and neuromuscular regeneration was found to be significantly reduced in aged rats as compared to young adult rats (Daniloff et al., 1986). The lower PSA-NCAM level in the aging nervous system has been linked to an age-associated decline in sialyltransferase activity, which ultimately leads to a decline in regenerative potential (Olsen et al., 1995). More recently, the effect of reduced sialylation in mice brains heterozygous for GNE, an essential enzyme for sialic acid biosynthesis (section “Sialic Acid Structure and Metabolism: An Overview”) was studied (Klaus et al., 2020). It was found that the GNE ± mice had hyposialylation in various brain regions along with less synapses in the hippocampus and lower microglial arborization at 6 months of age followed by an elevated neuronal loss at 12 months. Interestingly, the neuronal loss observed in these mice was not accompanied by a pro-inflammatory signature prototypic of inflammatory neurodegenerative disorders. During development, the microglial complement receptor 3 and the complement components C3 and C1q can regulate synaptic pruning and neuronal network maturation. Cross breeding of GNE ± mice with C3-deficient mice rescued the synaptic and neuronal loss along with a significant upregulation of the microglial marker, Iba1, indicating that the complement system plays a crucial role in mediating the neuronal loss in GNE ± hyposialylated mice. It has been postulated that GNE ± mice could mimic a physiological overall decrease in sialic acid with aging. This can cause a decline in masking of glycocalyx by sialic acids and subsequent exposure of underlying aminophospholipids could increase complement binding and thereby lead to complement mediated synaptic elimination and neuronal loss by microglia with age (Klaus et al., 2020). Furthermore, microglia have been found to be hypo-motile and chronically produce pro-inflammatory cytokines in an aged brain suggesting an impaired homeostatic functioning with age (Pluvinage et al., 2019). In order to elucidate the molecular mechanism underlying this impairment and to identify an age-related genetic modifier of microglia phagocytosis, Pluvinage et al. combined CRISPR-Cas9 screening in BV2 cells with RNA sequence analysis in mice brains. The study found that the expression of CD22, a Siglec typically found on B cells, was significantly increased in aged microglia and CD22-targeted deletion promoted phagocytosis in BV2 cells. In addition, CD22 was found to interact with cytidine monophosphate N-acetylneuraminic acid synthetase (CMAS), an important enzyme for sialic acid synthesis and PTPN6, a gene that encodes for SHP-1 for sialic acid-Siglec signaling. Targeted deletion of CMAS or PTPN6 or sialidase treatment phenocopied CD22 deletion in BV2 cells indicating involvement of sialic acid in CD22-mediated inhibition of microglial phagocytosis. Although there are other Siglecs that can also modulate microglial phagocytosis such as CD33, the study found that CD22 was the sole mouse Siglec upregulated with age where aged microglia expressed three times more CD22 than young microglia. Furthermore, phagocytotic clearance of myelin debris was upregulated with an anti-CD22 antibody injection compared to IgG control antibody injected mice. To identify the transcription functions of CD22 inhibition, aged mice were implanted with osmotic pumps to deliver anti-CD22 antibody into the CSF for a month and compared with an IgG control antibody infusion. The anti-CD22 antibody infusion was found to elevate the expression of homeostatic microglia genes such as Sall1, Mef2a, Tgfbr1, Il10ra, and P2ry13 and downregulate activated and disease related microglial genes such as Ccl3, Tspo, Lgals3, H2-K1, and Tnfsf13b, thereby restoring the transcriptional hallmarks of aging associated microglia damage. The study also addressed the effects of CD22 inhibition on age-associated cognitive dysfunctions. Aged CD22^–/–^ mice showed improved associative memory and spatial memory in the contextual fear conditioning and Y-maze test, respectively, as compared to aged WT mice. The same tests were also conducted in aged WT mice infused with anti-CD22 or IgG directly into the brain via an osmotic pump. The anti-CD22 infusions phenocopied the cognitive improvements exhibited by CD22^–/–^ mice indicating the specificity of CD22 in negatively affecting cognitive functions in the aging brain. Hence, CD22 is a mediator of anti-phagocytotic activity of sialic acid in the microglia and inhibition of this interaction reverses the decline in microglia functions and cognitive performance in aged brain (Pluvinage et al., 2019).

Furthermore, a gradual loss of learning and memory functions is one of the earliest and most predominant consequences of brain aging (Schnaar et al., 2014); however, a definite cause of the loss is still unclear. Long-term potentiation (LTP) is a form of neural plasticity affected by aging and has been established as a cellular correlate for learning and memory (Bergado and Almaguer, 2002; Barnes, 2003). PSA-NCAM plays a role in LTP in the hippocampus and significantly declines in the dentate gyrus with increasing age (Ní Dhúill et al., 1999). Two major types of N-methyl-D-aspartate (NMDA) receptor subunit, GluN2A and GluN2B, have been demonstrated to play opposite roles in LTP (Kochlamazashvili et al., 2010, 2012). An increase in the activation of GluN2B-containing NMDA receptors and a decrease in the activation of GluN2A-containing receptors have been linked to impairment in LTP. PSA-NCAM inhibits the binding of glutamate to extra synaptic GluN2B due to steric hindrance. As a result, GluN2A activation prevails and leads to LTP induction. In the absence of PSA-NCAM, GluN2B is activated at the expense of GluN2A, which in turn activates p38MAPK (mitogen-activated protein kinase), a major player in synaptic depression. This effect, in combination with the lack of activation of GluN2A, results in impaired LTP in aged brains (Kochlamazashvili et al., 2010, 2012). Moreover, studies have looked at ganglioside composition in brains of humans ranging from 20 to 100 years of age (Chiricozzi et al., 2020). Ganglioside level progressively reduced with age, reaching about 30% in centenary compared to 20-year-old subjects. In addition to the amount, ganglioside expression pattern was found to be altered as well. An increased level of b-series gangliosides, such as GD1b, and a decreased level of a-series gangliosides, including GM1 and GD1a, were reported. Ganglioside sub-series are defined based on the number of sialic acids linked to the galactose residue of the glucosylceramide core in gangliosides: sialic acid number equals 0 is the O-series; 1, the a-series; and 2, the b-series (Kolter, 2012). GM1 and GD1a are thus the major gangliosides associated with reduced sialic acid content in aging humans. A study aimed to identify the difference in the response between young and aged rats upon brain cold injury found an increase in brain sialic acid levels in young but not aged rats in response to injury (Uslu et al., 2004). Similarly, aging was found to affect the response to thermal stimuli where aged rats exhibited a longer latency in hot plate compared to younger rats which was reversed upon treatment with GM1 (Goettl et al., 2000). Intra-peritoneal injection of GM1 was found to increase the count and size of tyrosine-hydroxylase immunopositive neurons indicative of higher presynaptic dopaminergic indices in substantia nigra pars compacta of rats (Toffano et al., 1983). Furthermore, GM1 was shown to promote differentiation, protect against neuronal excitotoxicity, and facilitate response to neurotrophic factors (Chiricozzi et al., 2020). In summary, GM1 has been established as one of the major determinants of neuronal functions and its reduced biosynthesis is considered as one of the major causes for neuronal loss in aged brain.

## Sialylation in Alzheimer’s Disease

AD is a progressive neurodegenerative disorder that accounts for over 60% of the 46.8 million cases of dementia worldwide (Long and Holtzman, 2019). The nerve cell death causes memory loss and personality changes and disrupts one’s ability to carry out daily activities (Sundaram, 2017). The deposition of extracellular amyloid beta (Aβ) plaques and intraneuronal neurofibrillary tangles in the brain are the most prominent hallmarks of AD (Nasr et al., 2018). In addition, research aimed at developing a biomarker for AD has shown elevated levels of serum sialic acid in AD (Davis et al., 2009). Studies have also demonstrated various roles of sialic acid in the development of AD pathologies.

### Interaction of Gangliosides With Aβ

**FIGURE 2:**
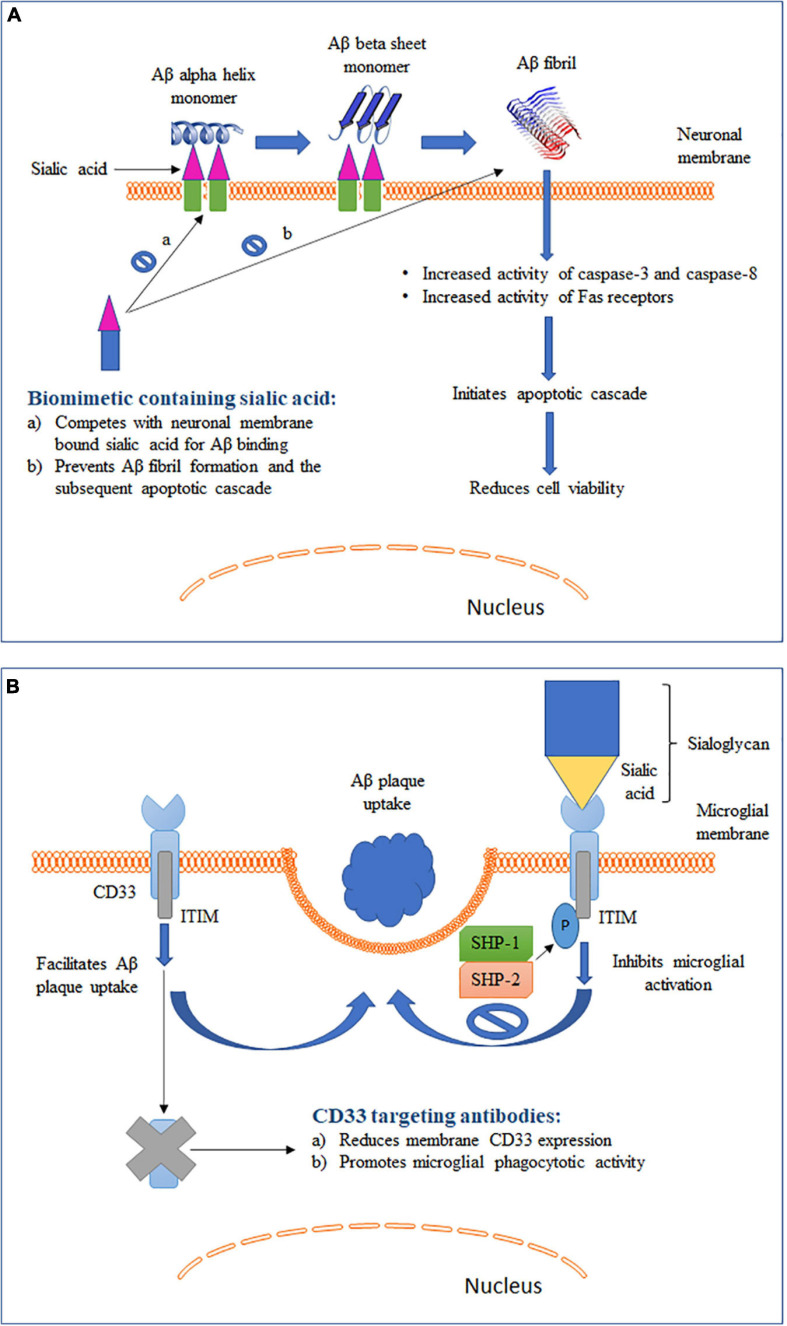
Schematic representation of two different roles of sialic acid in AD pathology along with their therapeutic intervention strategies. **(A)** Role of sialic acid in Aβ fibrillation and the associated cytotoxicity. **(B)** Role of sialic acid in CD33-mediated microglial functions.

Elucidation of the mechanism for conversion of soluble and non-toxic α-helix-rich Aβ into aggregated and toxic β-sheet–rich structures would help to understand the early pathogenesis of AD (Ariga et al., 2011). Studies have shown that Aβ1–40 can bind to gangliosides, particularly GM1, causing changes in the secondary structure of Aβ (Figure 2A). The rate of fibril formation of Aβ1–40 was accelerated with an addition of ganglioside-containing vesicles as compared to vesicles without gangliosides (Choo-Smith et al., 1997). In addition to GM1, Aβ 1-40 can also bind to several other gangliosides with the following descending order of binding strength: GQ1bα> GT1aα> GQ1b > GT1b > GD3 > GD1a = GD1b > LM1 > GM1 > GM2 = GM3 > GM4 (Ariga et al., 2001). It has been hypothesized that the ganglioside-bound Aβ self-associates on the surface of cell membranes and undergoes a conformation change to form a β-sheet–ordered structure (Chi et al., 2007). This serves as an initial step in the ganglioside-mediated fibrillation of Aβ. Although the exact mechanism is not clearly understood, possible involvement of electrostatic forces between negatively charged sialic acid and Aβ has been proposed (Ariga et al., 2011). A study reported a GM1-dependent Aβ fibril binding to murine endothelioma H-END cells (Bucciantini et al., 2012). The binding was highly localized to the cell membrane and resulted in an increase in cell death in a dose-dependent manner. However, cells pretreated with neuraminidase were unable to bind to Aβ fibrils. This sialic acid-mediated Aβ fibril binding was associated with an increase in the activity of caspase-8, an apoptotic mediator. Furthermore, binding of the Aβ fibril led to an increased activation of Fas receptors. Fas receptors are death receptors expressed on cell surfaces and cause apoptosis upon ligand binding. Similar observations were seen in another study where treatment of neuroepithelial cells with either GM1 or Aβ1–40 alone did not cause any change in cell viability (Yanagisawa et al., 2010). However, a significantly reduced viability was observed in cells cultured with both GM1 and Aβ1–40. The combination was associated with an increased expression of caspase-3, another critical mediator of apoptosis. These studies suggest that sialic acid-mediated Aβ binding and fibrillation causes cytotoxicity by triggering an apoptotic cascade. Yanagisawa et al. reported that Aβ1–42, and not Aβ1–40, binds strongly to GM1 in the AD brain (Yanagisawa et al., 1995). The bound Aβ1–42, termed ganglioside-bound Aβ (GAβ), could serve as an endogenous seed for Aβ accumulation. This observation was further validated in immunoprecipitation experiments using cerebral cortices from AD patients (Yanagisawa and Ihara, 1998). The immunoprecipitates obtained with Aβ1–42 N-terminal fragment monoclonal antibody also showed reactivity for cholera toxin-B subunit, a ligand highly sensitive and specific for GM1, suggesting Aβ1–42 and GM1 binding. GAβ exhibits unique properties such as high aggregation potential and changed immunoreactivity. These properties allowed GAβ to facilitate Aβ fibril formation in the brain. The increase in Aβ aggregation has been found to correlate positively with the increase in GM1 in neuronal membranes (Kurganov et al., 2004). Furthermore, removal of GM1 from the neuronal membranes was found to reduce Aβ-mediated cytotoxicity. Remarkably, an antibody targeting GAβ has been found to suppress Aβ deposition in the brain in mouse models of AD (Yamamoto et al., 2005; Yamamoto et al., 2008). In summary, these studies indicate that gangliosides play a crucial role in Aβ fibrillation and disrupting the ganglioside–Aβ interaction could significantly decrease Aβ deposition in AD brains.

### Regulation of CD33 by Sialic Acid

Late-onset Alzheimer’s disease (LOAD) is the most prevalent form of AD generally affecting individuals after the age of 65 (Griciuc et al., 2013; Wu et al., 2018). CD33 has been identified as one of the highly ranked genetic risk factors for the development of LOAD, with apolipoprotein (ApoE) ε4 at the top (Harold et al., 2009; Naj et al., 2011). Two different single nucleotide polymorphisms (SNPs) in CD33 have been reported: rs3865444C and rs3865444A. rs3865444C is a common allele (70% of the general population) and is associated with an increased expression of CD33 in the brain. Carriers of this allele had a greater likelihood of Aβ pathology and AD diagnosis (Griciuc et al., 2013; Malik et al., 2013). In contrast, rs3865444A, which is the minor A allele of CD33, is associated with reduced CD33 expression in the brain and has been found to be protective against AD (Raj et al., 2014).

CD33, also known as Siglec-3, is an inhibitory Siglec found to be expressed by microglia, monocytes and macrophages (Zhao, 2019). Binding of sialic acid-modified glycoproteins and glycolipids activates CD33 and leads to inhibition of cellular functions (section “Sialic Acid Physiochemical and Biological Properties” and Figure 2B). Studies have shown that microglia-mediated phagocytosis is dependent on the level of CD33 expressed by microglia (Griciuc et al., 2013; Jiang et al., 2014). Primary microglia cells derived from CD33 KO mice showed a higher Aβ uptake as compared to wild type (WT) cells (Griciuc et al., 2013). On the other hand, BV2 microglia cells that overexpressed CD33 had a significantly impaired Aβ uptake capacity. Circulating monocytes infiltrate the brain under pathological conditions, including AD (Jiang et al., 2014). Higher CD33 expression on the surface of monocytes was found to be associated with inhibition of Aβ phagocytosis. Overall, these findings point to an important role of CD33 expression in modulating Aβ clearance in the brain. To further identity the role of sialic acid in CD33-modulated Aβ clearance, BV2 microglia cells were transfected with a mutant CD33 (CD33^Δ^ ^V–Ig^), which lacked the sialic acid-interacting V-type immunoglobulin-like extracellular domain (Griciuc et al., 2013). The mutant CD33^Δ^ ^V–Ig^ was localized to BV2 cell plasma membrane and expressed at a level comparable to that of WT CD33. Inhibition of Aβ clearance by CD33 was eliminated in cells expressing mutant CD33^Δ^ ^V–Ig^, indicating that sialic acid interaction is needed for CD33 to modulate microglial Aβ uptake. These findings also provide support to a recently proposed hypothesis that Aβ plaque itself can dodge microglia mediated clearance with the help of sialic acid–CD33 interaction (Jiang et al., 2014). Aβ plaque often aggregates with sialic acid-containing glycoproteins and glycolipids and this aggregated form of Aβ can directly activate CD33 signaling and downregulate microglia mediated immune activation (Jiang et al., 2014). Hence, in addition to immunosuppression of microglia by increased CD33 expression, sialic acid–CD33 interaction can also efficiently mask microglia recognition and lead to Aβ accumulation in the brain independent of altered CD33 expression levels.

### PSA-NCAM in Adult Neurogenesis

Neurogenesis in an adult brain involves a complete neuronal development process, from proliferation, differentiation, and migration to synaptic integration and survival of the newly formed neurons (Gascon et al., 2010). Although the precise physiological relevance of adult neurogenesis is unclear, possible involvement in recovery from injury, learning and memory, as well as enhanced sensory discrimination of the olfactory bulb, has been demonstrated (Lledo et al., 2006). The subventricular zone (SVZ) below the ventricular walls and the subgranule layer (SGL) of the hippocampal dentate gyrus are the neurogenic niches of the adult brain (Haughey et al., 2002). These regions supply new neurons to the hippocampus and neocortex, the two major brain structures affected by AD. Aβ deposition has been observed in the SVZ and SGL and was found to disrupt proliferation and differentiation and induce apoptosis of neural progenitor cells (Kerokoski et al., 2001). Postnatal neurogenic niches are characterized by a prominent PSA-NCAM expression, which indicates that this molecule plays a role in neurogenesis (Cremer et al., 1994; Gascon et al., 2010). Hippocampal tissue analysis of AD patients showed an upregulation of PSA-NCAM with disease severity (Mikkonen et al., 1999, 2001; Jin et al., 2004). The hippocampal regions with increased PSA expression were also the regions where Aβ plaques, neurofibrillary tangles and neuronal loss occur and where neurons undergo remodeling (Jin et al., 2004). Consistent with this finding, acute injection of Aβ into rat hippocampus caused an increased PSA expression (Limón et al., 2011), which suggested an important role of PSA in neurogenesis-associated AD pathogenesis.

Studies have shown that genetic deletion of NCAM causes 30% reduction in the size of the olfactory bulb, with the overall brain size being decreased by 10% (Gascon et al., 2010). These effects were replicated by an injection of Endo-N, which indicated that the observed phenotype in NCAM-deficient animals was due to the absence of PSA-associated NCAM (Ono et al., 1994). Along with the reduced olfactory bulb size, an upregulated number of neuronal precursors were also seen in the SVZ-rostral migratory stream (RMS) in NCAM-deficient animals as compared to their WT littermates (Chazal et al., 2000). Neural stem cells present in the SVZ form neuroblasts that first migrate tangentially via RMS and then radially to the olfactory bulb (Gascon et al., 2010). It has been postulated that the accumulation of neuronal precursors in the SVZ-RMS is due to impaired migration to the olfactory bulb. Several studies have shown that PSA on NCAM are primarily responsible for the migration: (a) Migrating NCAM-positive cells have high PSA content (Kiss and Rougon, 1997; García-Verdugo et al., 1998); (b) functional inhibition of PSA on NCAM, by either enzyme-mediated removal or neutralizing antibody, with no changes to the core protein, was adequate to impair migration (Ono et al., 1994); and (c) deficiency of polysialyltransferases ST8SiaII and ST8SiaIV, the two enzymes responsible for PSA synthesis, caused abnormal tangential as well as radial migration during development (Angata et al., 2007). As discussed in section “Synaptic Development,” PSA reduces NCAM-mediated adhesive interaction, which is a property that has been postulated to allow for cell motility during neurogenesis (Ono et al., 1994). In addition to migration, PSA-NCAM has also been found to be important for the survival of newly formed immature neurons in primary cortical neuronal cultures (Ono et al., 1994). It has been shown that the Endo-N-mediated removal of PSA from NCAM drastically reduced the number of newly generated neurons. Similar effects were also seen when PSA was inhibited by specific antibodies and in cultures from NCAM-deficient mice. Although the exact mechanism by which PSA regulates survival of newly generated neurons is not clearly understood, but possible involvement of brain-derived neurotrophic factor (BDNF) signaling has been considered (Vutskits et al., 2001). BDNF is a member of the neurotrophin family, a group of secreted proteins that have a profound role in neuronal differentiation, growth and survival (Gascon et al., 2007). PSA-NCAM has been shown to increase the neuronal ability to respond to BDNF by facilitating the binding of BDNF to its receptor, tyrosine kinase receptor B (TrkB) (Muller et al., 2000). Removal of PSA from NCAM has been found to significantly reduce the level of TrkB phosphorylation and activation; however, how PSA facilitates BDNF-TrkB–induced signaling is unclear (Vutskits et al., 2001). Overall, PSA-NCAM is involved in the regulation of migration and survival of newly generated neurons and therefore is an important regulator of neurogenesis. In neurodegenerative conditions, such as AD, the ability of the brain to retain new neurons provides prospective cell replacement (Jin et al., 2004). This could result in beneficial consequences, especially in the brain regions disproportionately affected by AD, such as the hippocampus. Although there is no direct evidence in AD pathology, findings from spinal cord injury studies provide strong support (Mehanna et al., 2010). Subdural infusions of PSA glycomimetic was found to increase the number of monoaminergic axons and glutamatergic and cholinergic nerve terminals in the lumbar region of the spinal cord. Axon myelination and functional motor recovery were also found to be improved at areas in proximity to the injury site due to PSA-NCAM–mediated axonal outgrowth, branching, and defasciculation (section “Synaptic Development”) (Doherty et al., 1990; Becker et al., 1996). Although an increase in PSA in AD patients could be an attempt to compensate for the neuronal damage, cell loss continues to persist (Jin et al., 2004). Several hypotheses pointing to a limited restoration capacity of neurogenesis have been postulated to explain this gap. First, the extent or rate of cell loss could be too high for quantitatively significant replacement to occur. Second, the neurons produced may not convert into mature and fully functional neurons and thus become incapable of integration into the existing brain circuit. Third, the brain microenvironment in AD could be too toxic to facilitate survival of the newly generated neurons (Rapoport et al., 2002). Nevertheless, strategies aimed to increase and support neurogenesis could have a therapeutic value in AD.

### Alterations in Ganglioside and Sialic Acid Metabolism

Amyloid precursor protein (APP) processing by membrane-associated α-, β-, and γ-secretase is strongly dependent on membrane fluidity (Eckert et al., 2010; Bhattarai et al., 2020). Aβ has been shown to upregulate APP amyloidogenic processing by binding and reducing membrane fluidity, which increases its own production in HEK293 and SH-SY5Y cells, and this effect was postulated to be mediated by gangliosides due to their strong affinity to Aβ (Ariga et al., 2011). Multiple studies have reported altered ganglioside metabolism in AD and this alteration is predominantly manifested as decreased ganglioside levels in several brain regions (Brooksbank and McGovern, 1989; Crino et al., 1989; Kalanj et al., 1991). In addition, the pattern of alteration varied depending on the age of AD onset (Ariga et al., 2008). The amount of ganglioside was reduced by 58–70% compared to controls in gray matter and by 81% in frontal white matter in the brains of early-onset AD patients. However, a significant reduction was seen in the temporal cortex, frontal white matter and hippocampus in the brains of LOAD cases (Svennerholm and Gottfries, 1994). Similarly, brain analysis of individuals suffering from dementia of the Alzheimer type (DAT) also showed significantly reduced gangliosides as compared to the control group (Crino et al., 1989). The affected brain regions were the entorhinal and posterior cingulate, prefrontal cortex, nucleus basalis of Meynert, and visual cortex. Furthermore, studies have also reported alterations in ganglioside composition in AD brains where b-series gangliosides, including GD1b and GT1b, were decreased, whereas GT1a, GM1, GM2, and GD3 were increased in the frontal cortex (Brooksbank and McGovern, 1989; Kracun et al., 1992). Consistent with these findings, examination of cerebrospinal fluid of AD patients revealed that the total amount of gangliosides was comparable between the control and “probable AD” group; however, the distribution of ganglioside species was significantly different between the groups (Blennow et al., 1992; Kracun et al., 1992). The “probable AD” group had a higher level of GM1 and GD1a along with lower GT1b and GD1b levels. Although the mechanism for the observed changes in ganglioside composition in AD is unclear, these findings re-emphasize a crucial role of sialic acid-containing GM1 in forming GAβ and facilitating Aβ-membrane interactions (section “Interaction of Gangliosides With Aβ”). GM1 has also been shown to reduce the fluidity of sphingolipid-enriched membranes, which thereby favors amyloidogensis (Eckert et al., 2010). Apart from human studies, findings from mouse models of AD have been controversial. Sawamura et al. (2000) reported a lack of notable differences in the major brain gangliosides of mutant presenilin-2 mice, despite their having a remarkably elevated Aβ1–42 level. Furthermore, Bernardo et al. did not observe a significant alteration in a- or b-series gangliosides in the AD mouse model (APP with Swedish mutation and presenilin-1 with exon 9 deletion) as compared to WT (Bernardo et al., 2009). On the contrary, elevated levels of GM2 and GM3 and reduced GQ1b, GD1b, GT1a, and GD3 have been reported in the cortices of mice expressing human presenilin-1 and human APP with Swedish and London mutations (APP^SL^) (Barrier et al., 2007). Significantly increased levels of cortical GM2 and GM3 were also reported in APP^SL^ transgenic mice co-expressing a point mutation in presenilin-1 (Ariga et al., 2008). The disparities in these findings could be attributed to the different mouse models used for the studies (Grimm et al., 2013). Overall, the findings from human brains and selective mouse models of AD point to a prominent role of alteration in ganglioside metabolism in AD pathogenesis.

Untargeted metabolomics conducted on CSF of patients with MCI also revealed a higher sialic acid metabolism as compared to normal controls (Hajjar et al., 2020). Furthermore, association studies between discriminatory metabolites and disease phenotype using Spearman’s correlation analyses revealed that altered sugar metabolism was associated with elevated levels of tau and phosphorylated tau and reduced cognitive performance, cortical thickness, and hippocampal volume. This finding indicates that an impaired sugar regulation including sialic acid may occur years before AD is clinically manifested. It has been postulated that the increased sugar metabolism may in part be explained by a reduced brain glucose uptake, for instance, secondary to impaired glucose uptake transporter thereby leading to a higher CSF sugar level. Alternatively, central insulin resistance as reported in AD could lead to higher metabolic byproducts in the CSF (Hajjar et al., 2020). Based on these findings, another follow-up study hypothesized that glycan profile could also be altered in AD brains and therefore analyzed the N-linked glycan profile in the cortex and hippocampus in control and AD brains (Gaunitz et al., 2020). Two and four glycans in the cortex and hippocampus, respectively, showed different levels in AD brains as compared to controls. Strikingly, all the glycans that differed had similar structures: complex glycan with one sialic acid, a potential or confirmed bisecting N-acetylgluocasamine and at least one fucose. This finding provides support to previous propositions that N-glycans could serve as a relevant biomarker for AD and glycosylation is impacted in AD pathology, however, how this altered glycan profile affects AD pathology remains elusive (Gaunitz et al., 2020). Impaired neural glycosylation state has also been previously reported as a potential early event in neurodegenerative process in 1995, when it was found that the ST enzyme activity was significantly reduced in postmortem brain samples of AD patients as compared to age matched controls (Maguire and Breen, 1995). It was hypothesized that the altered neural ST activity could affect APP glycosylation state and the subsequent production of Aβ. This notion has since been supported by a study conducted by Annunziata et al. that investigated the functions of NEU1 and lysosomal exocytosis in amyloidogensis in mouse brains with NEU1-targeted deletion (Annunziata et al., 2013). As discussed in section “Sialic Acid Structure and Metabolism: An Overview,” NEU1 induces sialyglycoconjugates catabolism by eliminating their terminal sialic acids. NEU1 also regulates lysosomal exocytosis by limiting the sialic acid content of lysosomal-associated membrane protein-1 (LAMP1). LAMP1 facilitates the recruitment of lysosomal pool to the plasma membrane and the subsequent release of luminal content extracellularly and without NEU1, hypersialylated LAMP1 increases lysosomal recruitment and lysosomal exocytosis. NEU1^–/–^ mice was found to exhibit accumulation of oversialylated APP in endolysosomes, a novel substrate of NEU1. Furthermore, the endolysosomal APP was proteolytically cleaved to produce Aβ that was released extracellularly by exacerbated lysosomal exocytosis. Remarkably, an intracranial injection of NEU1 to the 5XFAD mouse model of AD reduced the number of Aβ plaques and Aβ peptide levels. Therefore, NEU1 was identified as a risk factor for developing AD-like amyloidosis (Annunziata et al., 2013).

## Sialylation as a Therapeutic Target for AD

### Sialic Acid to Reduce Aβ Toxicity

Binding of Aβ to sialic acid on gangliosides on neuronal membranes has been shown to promote amyloidosis and induce cytotoxicity in AD (section “Interaction of Gangliosides With Aβ” and Figure 2A). Furthermore, inhibition of synthesis or enzymatic removal of membrane-associated sialic acid was found to be neuroprotective against Aβ-induced toxicity (Patel et al., 2006). These findings prompted the development of strategies to disrupt the cytotoxic interaction between Aβ and sialic acid and by such strategies to reduce AD pathology (Dhavale and Henry, 2012). Kakio et al. (2001) reported that the affinity of Aβ to cell membrane increases when sialic acids are clustered on the cell surface. This finding led to the hypothesis that a membrane mimic with similar sialic acid structure could compete with physiological membrane for Aβ binding (Patel et al., 2006). In support of this hypothesis, a sialic acid-conjugated dendrimer was found to bind and sequester Aβ, thus rendering it unavailable for cell interaction, which led to improved cell viability (Patel et al., 2007). Furthermore, Yin et al. (2015) synthesized a selenium nanoparticle modified with sialic acid and alternative-B6 peptide conjugation (B6-SA-SeNPs). Selenium nanoparticles are widely used because they exhibit very low toxicity and possess anti-oxidative properties. B6 peptide allowed the compound to show high blood-brain barrier permeability (Liu et al., 2013). More importantly, sialic acid on the nanoparticle inhibited Aβ fibrillation and reduced Aβ toxicity in a dose-dependent manner in two different cell models. Moreover, the nanoparticle caused the preformed Aβ fibrils to disaggregate into non-toxic oligomers, making it a promising therapeutic agent to reduce AD pathogenesis. Similarly, inhibition of Aβ-mediated toxicity in SH-SY5Y cells was obtained by sialic acid-conjugated chitosan, a polysaccharide containing D-glucosamine and N-acetyl-D-glucosamine (Dhavale, 2009). Overall, these studies show that a biomimetic compound containing sialic acid structures similar to those expressed on neuronal membranes can compete and prevent cell surface Aβ binding and associated cellular damage, making it a promising strategy to reduce the development of Aβ pathology in AD (Figure 2A).

### Inhibition of CD33–Sialic Acid Interaction

With failure of drugs targeting Aβ plaques and neurofibrillary tangles and identification of multiple AD risk genes exclusively expressed by microglia (Bellenguez et al., 2020), alternative microglia-based therapies have become a more recent focus of AD drug discovery (Spangenberg and Green, 2017). Such therapies, while facilitating phagocytotic clearance of Aβ plaques and hyperphosphorylated tau, could also restore microglial phenotype to a healthy and functional state. The protective CD33 allele (rs3865444A) causes reduced expression of CD33 and is associated with lower AD risk (Zhao, 2019). Consequently, strategies targeting CD33 have been aimed to reduce CD33 expression and prevent CD33-mediated inhibition of microglial Aβ phagocytosis (Figure 2B). Gemtuzumab ozagamicin and lintuzumab are two CD33 antibodies that have been extensively tested in patients suffering from acute myeloid leukemia (AML) (Jurcic, 2012). These antibodies were found to reduce the expression of CD33 on the cell surface of monocytes. Lintuzumab also reduced the expression of CD33 by 50 and 80% in non-differentiated U937 cells and differentiated U937 cells, respectively (Zhang et al., 2016). These studies, along with the well-established safety profile in clinical trials for AML, have positioned lintuzumab as one of the top therapeutic candidates to be repurposed for AD. Antibodies targeting CD33 are able to reduce cell surface CD33 expression by inducing internalization and degradation (Malik et al., 2015). It is important to note that both of these antibodies act specifically against the domain encoded by exon 2 that mediates sialic acid binding to CD33 (Jurcic, 2012). Therefore, they work by specifically reducing the levels of sialic acid-binding CD33 isoforms (Malik et al., 2015). Alternatively, small molecular inhibitors of sialic acid–CD33 interaction have also been considered to be a suitable CD33 intervention strategy (Wes et al., 2016). However, targeting CD33 with small molecules has posed several challenges (Zhao, 2019). First, the sialic acid-binding region in CD33 is very flat, with no binding pockets. Second, this region has high polarity and thus requires a polar small molecule inhibitor (Varki and Angata, 2006). Polar molecules are very unlikely to permeate the blood-brain barrier, thus making the inhibition of sialic acid–CD33 interaction challenging. Therefore, a non-polar allosteric site that can be approached by small molecules to efficiently interrupt sialic acid binding to CD33 might be a better target (Zhao, 2019). Recent discovery of the 3D structure of CD33 and its binding domains will allow for identification of various allosteric modulators (Miles et al., 2019). In summary, CD33-based immunotherapy and sialic acid–CD33 small molecule inhibitors represent two promising avenues in the development of microglia-based AD therapeutics.

### Monocyte-Derived Activating Siglecs

The presence of Aβ plaques in the brain induces microglial activation and triggers an inflammatory response (Koenigsknecht and Landreth, 2004). Acutely activated microglia can efficiently cause Aβ phagocytosis and prevent plaque formation. However, suppression of such microglial activation inhibits Aβ phagocytosis and leads to Aβ deposition in the brain. Although inhibitory Siglecs with ITIM and ITIM-like signaling motifs are the major form of Siglecs, activating Siglecs also exist (Siddiqui et al., 2019). Activating Siglecs possess immunoreceptor tyrosine-based activation motif (ITAM), facilitate MAPK signaling, and cause immune cell activation. In other words, activating Siglecs when bound by their ligands can promote microglial phagocytic functions. Studies have shown that, when transferred to the brain, the bone marrow-derived monocytes induced efficient Aβ phagocytosis (Malm et al., 2005; Simard et al., 2006). Monocytes express several activating Siglecs, such as Siglec 14 (Fong et al., 2015) and Siglec 15 (Takamiya et al., 2013) in humans. It has been postulated that, since the majority of microglial Siglecs are inhibitory, exposure of sialylated plaques to monocytes can allow sialic acid to bind to monocyte-resident activating Siglecs (Salminen and Kaarniranta, 2009). This in turn activates the immune response and induces phagocytosis and clearance of Aβ plaques. With promising results in mouse models of AD, this approach has the potential to limit Aβ plaque formation and deposition in AD.

### GM1 as a Peripheral Sequester of Aβ

One of the therapeutic approaches tested for AD treatment is peripheral administration of anti-Aβ antibodies to reduce Aβ load in the brain (Matsuoka et al., 2003). These treatments have been shown to alter Aβ dynamics and lead to Aβ efflux from the brain to the circulation, a process that is referred to as the “peripheral sink” effect (DeMattos et al., 2002). However, due to adverse patient response associated with Aβ-based immunotherapy, clinical trials were suspended (Matsuoka et al., 2003). Nonetheless, this concept has encouraged researchers to identify alternative compounds that can bind to Aβ in the periphery. As discussed in section “Interaction of Gangliosides With Aβ,” sialic acid-mediated binding between GM1 and Aβ forms GAβ and promotes Aβ deposition. Prompted by this finding, Matsuoka et al. (2003) examined the effects of peripheral administration of GM1 in APP/PSEN1 mouse model of AD. The treatment induced a significant decrease in both Aβ1–40 and Aβ1–42 aggregation in the brain with a parallel increase in plasma Aβ levels. Furthermore, the GM1-bound Aβ could no longer cross the blood-brain barrier and form plaques centrally. In summary, peripheral administration of GM1 can potentially sequester Aβ in the plasma and reduce brain amyloidosis. This approach has paved the way to develop novel therapeutics that are not limited by adverse immune response or brain permeability in AD.

In addition, a monoclonal antibody, 4396C, that targets GAβ was developed (Hayashi et al., 2004). This antibody was found to inhibit the aggregation of Aβ1–40 and Aβ1–42 *in vitro* by binding to GAβ. Furthermore, peripheral administration of the antibody was found to significantly reduce Aβ accumulation in the brain of transgenic mice expressing mutant human APP gene (Yamamoto et al., 2005). Overall, these findings support the development of GM-1-based therapeutics to inhibit Aβ aggregation in AD.

## Conclusion and Future Perspectives

Sialic acids represent a diverse family of sugars that possess a 9-carbon backbone and are mostly found as terminal residues in glycans of glycoconjugates. Sialic acids have been shown to play a variety of roles in human physiology and pathophysiology, ranging from kidney filtration to airway lubrication to cancer progression. The highest levels of sialic acids are found in the brain, where they are expressed mainly in gangliosides and PSA-NCAM. These two sialic acid carriers have been shown to regulate important brain functions, including axon myelination, synapse development and transmission, and modulation of microglial homeostasis. Age-associated loss of sialic acid in the brain has been demonstrated to negatively affect the regenerative potential of neuronal fibers, neural plasticity and microglial phagocytosis. Moreover, a decrease in ganglioside levels has been linked to increased neuronal loss in aged brains. In addition to aging, sialic acid has also been indicated to play important roles in AD pathogenesis. While gangliosides primarily affect Aβ accumulation and deposition, PSA-NCAM deficiency has been associated with reduced brain repair capabilities in AD. Furthermore, the ability of sialic acid itself to serve as a ligand for Siglec enables it to alter microglial functions and axon myelination. Although a detailed mechanism for several sialic acid-mediated functions remains to be known, the existing knowledge has provided a foundation to develop sialic acid-based therapeutics in AD. Targeting sialic acid has so far shown promising results with its ability to downregulate Aβ plaque formation. However, AD is a heterogeneous disease with complicated pathophysiology. Over the past two decades, extensive efforts have been made to reduce the levels of Aβ and its aggregation and increase its clearance from the brain (Long and Holtzman, 2019). Unfortunately, these efforts have failed to deliver a cognitive improvement in clinical trials. More recently, the presence of sustained inflammation mediated by microglia has been recognized as a core pathology in AD, leading to the focus of microglia-based strategies in AD drug discovery (Kinney et al., 2018). Despite the importance of Siglecs in mediating microglial activity and inflammation, the downstream ITIM and ITAM signaling is not fully understood (Linnartz et al., 2010). In addition, although the role of a small number of Siglecs has been defined, overall, the Siglec family remains underexplored in the context of neuroinflammation (Siddiqui et al., 2019). Species-specific expression and lack of monoclonal antibodies against Siglecs have further contributed to this gap. Moreover, confounding findings have been presented where Siglecs can play either a preventive or a causative role in AD, warranting further studies to bring clarity to these inconsistencies. There is also a pressing need for a better understanding of the neuroimmune pathways and responsible molecular players involved. Although research on gangliosides and PSA-NCAM in the brain has been extensive, approaches to explore novel functions of the sialo residues are necessary. Furthermore, examination of the biosynthesis pathway would allow us to understand how sialic acid levels are altered in different pathologies. Studies using mouse models with targeted deletion of NCAM or gangliosides have been crucial to understand the functional role of these sialoglycans. However, it should be noted that such deletions can remove both the sialylated and the unsialylated forms of the molecule, which can result in changes not specific to the sialylated form but instead caused by entirely wiping off the molecule. Inconsistent results could also arise with the use of cancer cell lines for sialic acid studies. As discussed in section “Sialic Acid Physiochemical and Biological Properties,” cancer cells have a notorious reputation for utilizing sialic acids to their benefit such that hypersialylation is used to identify cancer stage and disease prognosis. Therefore, primary cell culture or tissue preparation could be better models for studying sialic acid–Siglec interactions. In addition, there are discrepancies in studies looking at Aβ and sialic acid binding and aggregation. Some of the studies have utilized Aβ1–40 peptide, whereas others have utilized Aβ1–42 peptide. Although they differ by only two amino acids, they have significant differences in metabolism, physiological functions, toxicities, and mechanism of aggregation (Qiu et al., 2015). This could be a causal factor for the discrepancy observed when trying to identify the role of sialic acid binding in neuronal toxicity.

In addition, studies on proteins relevant to AD pathogenesis could provide an insight into the role of sialylation in AD. Clusterin (CLU), also known as apolipoprotein J, is the third most prominent genetic risk factor for LOAD (Herring et al., 2019). Translation at exon 2 forms mature secreted CLU preprotein that is targeted to endoplasmic reticulum for glycosylation. CLU bearing six N-linked glycosylation is then further transported to Golgi for glycan modification and sialylation. Studies that sought to identify a novel biomarker for chronic ethanol consumption found CLU to be highly sialylated (26–28 moles of sialic acid residues per mole of CLU) as compared to 4 moles of sialic acid in the classic biomarker, carbohydrate-deficient transferrin (Torrente et al., 2012). Chronic ethanol exposure reduces the level of liver dolichol, a crucial mediator in the first step of N-linked glycosylation (Burda and Aebi, 1999). Reduced N-linked glycosylation causes CLU to lose its sialic acid and regain it upon alcohol abstinence (Lakshman et al., 2001; Ghosh et al., 2002; Javors and Johnson, 2003; Wurst et al., 2012). Therefore, the presence of sialic acid on CLU has been established as a reliable biomarker for chronic alcohol consumption. Despite these findings, the functional role of sialic acid present on CLU is unknown. In addition, ApoE ε4 and desmoglein-2, two other known LOAD risk genes, are also sialylated (Xu et al., 1999; Sugano et al., 2008; Giri et al., 2016; Debus et al., 2019). Additional studies are needed to understand the functional role of sialic acid residues on these proteins. Filling in these gaps would contribute to a better understanding of the role of sialylation and Siglec in AD. This could also allow us to explore the clinical potential of modulating sialic acid interactions in AD intervention.

## Author Contributions

Both authors wrote the manuscript.

## Conflict of Interest

The authors declare that the research was conducted in the absence of any commercial or financial relationships that could be construed as a potential conflict of interest.
